# Unique morphology and photoperiodically regulated activity of neurosecretory canopy cells in the pond snail *Lymnaea stagnalis*

**DOI:** 10.1007/s00441-023-03799-x

**Published:** 2023-07-07

**Authors:** Yoshitaka Hamanaka, Sakiko Shiga

**Affiliations:** Laboratory of Comparative Neurobiology, Graduate School of Science, Osaka University, Machikaneyama-Cho 1-1, Toyonaka, Osaka 560-0043 Japan

**Keywords:** Caudo-dorsal cell, Intracellular recording, Lateral lobe, Neurosecretory cell, Photoperiodism

## Abstract

The pond snail *Lymnaea stagnalis* exhibits clear photoperiodism in egg laying; it lays more eggs in long-day conditions than in medium-day conditions. A key regulator of egg laying is neurosecretory caudo-dorsal cells (CDCs) producing an ovulation hormone in the cerebral ganglia. Paired small budding structures of the cerebral ganglia (viz. the lateral lobe) also promote egg laying in addition to spermatogenesis and maturation of female accessory sex organs. However, it remains unknown which cells in the lateral lobe are responsible for these. Previous anatomical and physiological studies prompted us to hypothesize that canopy cells in the lateral lobe modulate activity of CDCs. However, double labeling of the canopy cell and CDCs revealed no sign of direct neural connections, suggesting that activity of CDCs is regulated either humorally or through a neural pathway independent of canopy cells. In addition, our detailed anatomical re-evaluation confirmed previous observations that the canopy cell bears fine neurites along the ipsilateral axon and extensions from the plasma membrane of the cell body, although the function of these extensions remains unexplored. Furthermore, comparison of electrophysiological properties between long-day and medium-day conditions indicated that the canopy cell’s activity is moderately under photoperiodic regulation: resting membrane potentials of long-day snails are shallower than those of medium-day snails, and spontaneously spiking neurons are only observed in long-day conditions. Thus, canopy cells appear to receive photoperiodic information and regulate photoperiod-dependent phenomena, but not provide direct neural inputs to CDCs.

**Supplementary Information:**

The online version contains supplementary material available at 10.1007/s00441-023-03799-x.

## Introduction

Most animals in temperate zones or higher latitudes adapt their development and reproduction to the annually cycling environment. They promote development and/or reproduction in favorable seasons, whereas they cease such activities under adverse conditions to undergo diapause, hibernation, or migration (Nelson et al. 2010). Such phenotypical changes, which require days, weeks, or even months to complete, are generally coupled to the neuroendocrine system. Thus, for animals, it is crucial to keep track of seasonal cues and anticipate upcoming seasons. Of various environmental cues, it is a length of day or night (i.e., photoperiod) that most animals rely on to sense approaching seasons because the cyclical change is consistent from year to year. The response to photoperiod is referred to as photoperiodism, and many invertebrates and some vertebrates are capable of processing photoperiod for seasonal adaption (Nelson et al. 2010; Dardente et al. 2019).

Since its discovery in the aphid (Marcovitch 1923), neuroendocrine mechanisms underlying animal photoperiodism have been vigorously addressed not only in Arthropoda such as insects but also in Chordata including mammals, birds, and fishes. However, we largely lack studies in other phyla, which have been waited for general understanding of the neuroendocrine mechanism of photoperiodism. Molluskan species are suitable candidates because they exhibit photoperiodism in several aspects of physiological phenomena, involving reproduction, growth, temperature tolerance, and learning capabilities (Numata and Udaka 2010; Hussein et al. 2020). Molluskan species are favorable research subjects because of their simple central nervous system (CNS). For example, the CNS of the pond snail *Lymnaea stagnalis* comprises ~ 15,000 to 20,000 neurons (Geraerts et al. 1991; Kemenes and Benjamin 2009), as little as one-tenth of the number of neurons constituting the brain of the fruit fly *Drosophila melanogaster* (~ 200,000 neurons) (Raji and Potter 2021). Therefore, it should be feasible to understand the mechanism in invertebrates underlying photoperiodic regulation of the neuroendocrine system at the level of local neuronal circuits within the CNS.

The hermaphroditic pond snail *L. stagnalis* exhibits clear photoperiodism in egg-laying behavior: long-day conditions promote egg laying, while short- to medium-day conditions retard it (Bohlken and Joosse 1982; Hamanaka and Shiga 2021; Kitai et al. 2021). Interestingly, the reproductive gland, viz. ovotestis, is known to develop irrespective to photoperiods the pond snail underwent (Kitai et al. 2021). Egg-laying activity seems to be causally linked to the change of excitability of neurosecretory caudo-dorsal cells (CDCs) in the cerebral ganglia that produce and release an ovulation hormone, viz. CDC hormone-I (CDCH-I) (Hamanaka and Shiga 2021). While snails in long-day conditions have been demonstrated to express more *cdch* mRNA than those in medium-day conditions (Kitai et al. 2021), we still do not have a firm grasp on what causes the change of the electrophysiological properties and *cdch* expression in CDCs. Exploring and characterizing other neurosecretory cells (NSCs) that are photoperiodically controlled should lead us to comprehend the central photoperiodic mechanism.

In *L. stagnalis*, in addition to the CDCs, the lateral lobe that is a small budding structure on a lateral side of the cerebral ganglion plays an important role in egg-laying activity (Geraerts 1976a). The lobe promotes egg laying and is believed to function as a coordinating center to balance growth with reproduction because the body growth is enhanced while the ovipository activity is attenuated in snails whose lateral lobe is cauterized; body growth is accelerated via other neurosecretory light green cells (LGCs) in the cerebral ganglia (Geraerts 1976a, b). The lateral lobe is also known to promote both the spermatogenesis and maturation of female accessory sex organs (Geraerts 1976a). Nevertheless, it has not yet been elucidated which cells in the lateral lobe are responsible for these reproductive processes.

The lobe houses at least three distinct types of neurosecretory cells (i.e., one canopy cell, two droplet cells, and some bipolar cells) as well as one follicle gland (Lever and Joosse 1961; Brink and Boer 1967; Geraerts 1976a). Among them, the canopy cell is the largest NSC expressing molluskan insulin-related peptides (MIPs) (van Minnen et al. 1989; Meester et al. 1992; Smit et al. 1992) although other functions are still elusive. Intriguingly, a quantitative electron microscopic (EM) study implied that the secretory activity of the canopy cell is photoperiodically regulated because the volume of the rough endoplasmic reticulum and Golgi zone indispensable for production of secretory materials is significantly larger in long-day conditions than in short-day conditions (van Minnen and Reichelt 1980). Accordingly, it is plausible that the canopy cell is involved in photoperiodic regulation of egg laying. These reports prompted us to investigate the activity of the canopy cell to examine if the activity is itself photoperiodically regulated, since the secretory activity of neurons is generally coupled to electrical activity. Further, the lateral lobe has been demonstrated to stimulate activity of CDCs (Roubos et al. 1980), which encouraged us to re-evaluate the detailed arborization patterns of the canopy cell and its potential neural connection with CDCs.

In the current study, we measured the electrical activity of the canopy cell, followed by anatomical inspection of the recorded cells to confirm the nature of recorded cells, and we compared the electrophysiological properties between long-day and medium-day conditions.

We conclude that the canopy cell is under moderate photoperiodic control, which reveals a positive correlation between ultrastructural changes of the subcellular organelle (van Minnen and Reichelt 1980) and electrophysiological properties (the present study). Our study also revealed detailed anatomical characteristics of a single canopy cell, and that there is no clear-cut evidence of direct neural connection between the canopy cell and CDCs. Therefore, the canopy cell might control CDCs via a humoral pathway, or alternatively another cell in the lateral lobe might be responsible for the regulation of CDCs.

## Materials and methods

### Animals

The pond snail *Lymnaea stagnalis* was reared according to a method previously described (Hamanaka and Shiga 2021). To collect egg masses, 3 to 5 adult snails fed on Japanese mustard spinach (*Brassica rapa var. perviridis*) were kept in long-day conditions (16 h light:8 h darkness, 16L8D) at 20 ± 1 °C. The collected egg masses were maintained in medium-day conditions (12 h light:12 h darkness, 12L12D) at 20 ± 1 °C until hatching. Fifteen newly hatched juveniles were, as a group, harvested and transferred into a transparent plastic cup with a lid (diameter 100 mm, height 70 mm) containing fresh water, and then maintained in 12L12D at 20 ± 1 °C. Snails that reached 13 weeks old were individually transferred to individual plastic cups on the final day of the 13 weeks old; half were reared in 16L8D at 20 ± 1 °C while the rest in 12L12D at 20 ± 1 °C. Water and food were exchanged twice a week, and the number of egg masses each snail laid was counted.

### Intracellular recording and dye filling

For intracellular recording, 25- or 26-week-old adult snails were used. The snails were anesthetized with 0.2 M MgCl_2_ in fresh water for 10–30 min at room temperature (22–26 °C), and then the soft body was pinned down on a dissecting dish. The skin was longitudinally cut with a pair of scissors and opened with insect pins in order to expose the CNS. The lateral lobe housing the canopy cell was carefully desheathed with a sharp tungsten needle, and a piece of filter paper soaked with 10% actinase in *Lymnaea* Ringer solution (Sunada et al. 2014) was placed over the lateral lobe for 3 min at room temperature to facilitate microelectrode penetration, followed by rinsing with Ringer solution several times. The CNS without the buccal ganglia was dissected out of the body, placed on a Petri dish, and submerged in Ringer solution. An Ag–AgCl reference electrode was placed near the tissue. For intracellular recording from the cell body of the canopy cell, the tip of a glass microelectrode was filled with 5% Lucifer yellow (L0259, Sigma-Aldrich, St. Louis, MO, USA) in 1 M LiCl, and the rest of the electrode supplied with 1 M LiCl, to give a resistance value of 30–65 MΩ. Electrical signals were amplified through a microelectrode amplifier (MEZ-8301, Nihon Kohden, Tokyo, Japan) and monitored on an oscilloscope (CS-4026, Kenwood, Tokyo, Japan). The data were digitalized through Axon Digidata 1550 (Molecular Devices, San Jose, CA, USA) at a sampling rate of 20 kHz, stored on a personal computer, and analyzed by AxoScope ver. 10.4 (Molecular Devices). Recording was conducted during the daytime (ZT1 to ZT9) at room temperature.

After recording, individual neurons were labeled by iontophoresis with Lucifer yellow. Hyperpolarizing direct current (5–7 nA) was injected for 10–20 min through the microelectrode using an electronic stimulator (SEN-3301, Nihon Kohden). After dye injection, the cerebral ganglia were cut off, and subsequently fixed with 4% paraformaldehyde (PFA) in 0.067 M phosphate buffer (PB, pH 7.2) overnight at 4 °C, followed by several washes with PB. The tissue was then dehydrated with a graded ethanol series and cleared with methyl salicylate for confocal microscopy. For some preparations, cerebral ganglia containing a Lucifer yellow-filled canopy cell were further processed for immunohistochemistry with a polyclonal antiserum raised against a caudo-dorsal cell hormone I (CDCH-I) as described below.

### Estimation of electrophysiological properties of a canopy cell

We determined the following seven properties from recordings: (i) resting membrane potential, (ii) threshold current necessary to elicit at least one action potential, (iii) membrane potential at which the first spike was initiated, (iv) threshold voltage, (v) spike duration, (vi) spike height, and (vii) input resistance. A positive current was gradually injected through the microelectrode using the electronic stimulator with 0.1-nA steps until injected current elicited action potentials. Subsequently, negative current was injected in the same manner until the membrane potential becomes unstable (up to – 3 nA at maximum). Input resistance was estimated by calculating the linear regression from the current–voltage relation created by both depolarizing and hyperpolarizing current steps. The reported value was obtained by subtracting the electrode resistance from regressed figure. Resting membrane potential value corresponds to that at the initiation of recording. Threshold voltage was calculated as the minimum depolarization from the resting membrane potential necessary to elicit at least a single action potential, and thus that of spontaneously spiking neurons was determined to be zero. For non-spontaneously spiking cells, the spike duration and height were calculated from the first spike that was triggered by injection of positive current. In spontaneously firing cells, the duration and height were calculated from the 2nd or 3rd spike after recording started. The spike duration was calculated as the value at half amplitude.

**Table 1:** Comparison of electrophysiological properties of canopy cells between medium-day and long-day conditions

	Threshold current (nA) ^1^	Membrane potential at 1st spike initiation (mV) ^2^	Spike duration (ms) ^1^	Spike height (mV) ^2^	Input resistance (MΩ) ^2^
12L12D, 20 °C (*n* = 16)	0.18 ± 0.075 ^*N.S*^	− 37.6 ± 10.1 ^*N.S*^	17.3 ± 6.8 ^*N.S*^	70.7 ± 12.4 ^*N.S*^	125.1 ± 43.2 ^*N.S*^
16L8D, 20 °C (*n* = 15)	0.17 ± 0.12	− 36.0 ± 11.2	19.9 ± 6.4	65.9 ± 15.6	115.2 ± 41.5

Note that all values are expressed as mean ± standard deviation. Spike duration was determined at half-amplitude. For details of the calculation of each parameter, see “Materials and methods” section. Respective parameters were statistically compared between medium-day (12L12D) and long-day (16L8D) conditions at 20 °C

^1^, Mann–Whitney *U* test; ^2^, unpaired two-tailed *t*-test; ^*N.S.*^, no significant difference

### Immunofluorescent labeling

Immunofluorescent labeling was performed according to previous studies (Hamanaka et al. 2012, 2016). The cerebral ganglia containing a Lucifer yellow-filled canopy cell were fixed with 4% PFA in 0.067 M PB overnight at 4 °C. After washing in PB, the tissues were embedded in gelatin/albumin mixture and post-fixed in 7–8% formaldehyde in PB overnight. The post-fixed tissues were sectioned in a horizontal plane at a thickness of 40 μm with a vibrating blade microslicer (DTK-3000, Dosaka, Kyoto, Japan) and washed in PB. Non-specific binding sites were blocked with 5% normal goat serum (NGS) in 0.01 M phosphate-buffered saline containing 0.5% Triton X (PBST, pH 7.4) for 1 h, and then incubated with a rabbit polyclonal antiserum against CDCH-I (1:500) in PBST containing 5% NGS for 3 days at 4 °C. After washing in PBST, the sections were incubated in a goat anti-rabbit IgG conjugated to Cy3 (1:200; Jackson ImmunoResearch, West Grove, PA, USA) in PBST containing 5% NGS for 2 days at 4 °C. The sections were then washed with PBST and PB, collected on gelatin-coated slide glasses, and mounted beneath a cover slip in Vectashield (H-1000; Vector, Burlingame, CA, USA). Antigen information of anti-CDCH-I and the specificity in *L. stagnalis* has been described elsewhere (Hamanaka and Shiga 2021).

### Photography and three-dimensional reconstruction of neurons

The canopy cell in the lateral lobe was digitally imaged with an optical microscope (BX51, Olympus, Tokyo, Japan) equipped with a CCD camera (DP72, Olympus). Tissues labeled with dye injection and/or immunofluorescence were imaged using a confocal laser scanning microscope (LSM710, Carl Zeiss, Jena, Germany) equipped with Plan-Apochromat × 10/0.45 M27, EC Plan-Neofluar × 20/0.50 M27, and Plan-Apochromat × 63/1.40 Oil DIC M27 (Carl Zeiss). In preparations labeled with Lucifer yellow alone, Lucifer yellow was excited with an Ar laser at 458 nm and viewed through a 477- to 685-nm band-pass filter. In those double-labeled with Lucifer yellow and Cy3, Lucifer yellow was excited with an Ar laser at 458 nm and viewed through a 477- to 559-nm band-pass filter, while Cy3 was excited with a diode-pumped solid-state laser at 561 nm and viewed through a 565- to 727-nm band pass filter. Confocal images were acquired at *z*-axis intervals of 0.41–3.35 μm and a resolution of 1024 × 1024 pixels. Confocal images in whole-mount preparations were compressed stacks of 92–457 optical sections while those in micro-sections 15 and 88 optical sections. The size, contrast, and brightness were adjusted using Photoshop 2021 (Adobe Systems Inc., San Jose, CA, USA) and Corel Draw 2018 (Corel Corporation, Ottawa, ON, Canada).

For three-dimensional (3D) reconstruction, LSM files acquired by LSM710 were processed with image processing software (Amira v. 2019, Thermo Fisher Scientific, Waltham, MA, USA). Profiles of each Lucifer yellow-filled neuron and outline of the lateral lobe were manually segmented. Generate surface and surface view were applied for 3D reconstruction of these.

## Results

### Gross anatomy of a canopy cell

**Fig. 1:**
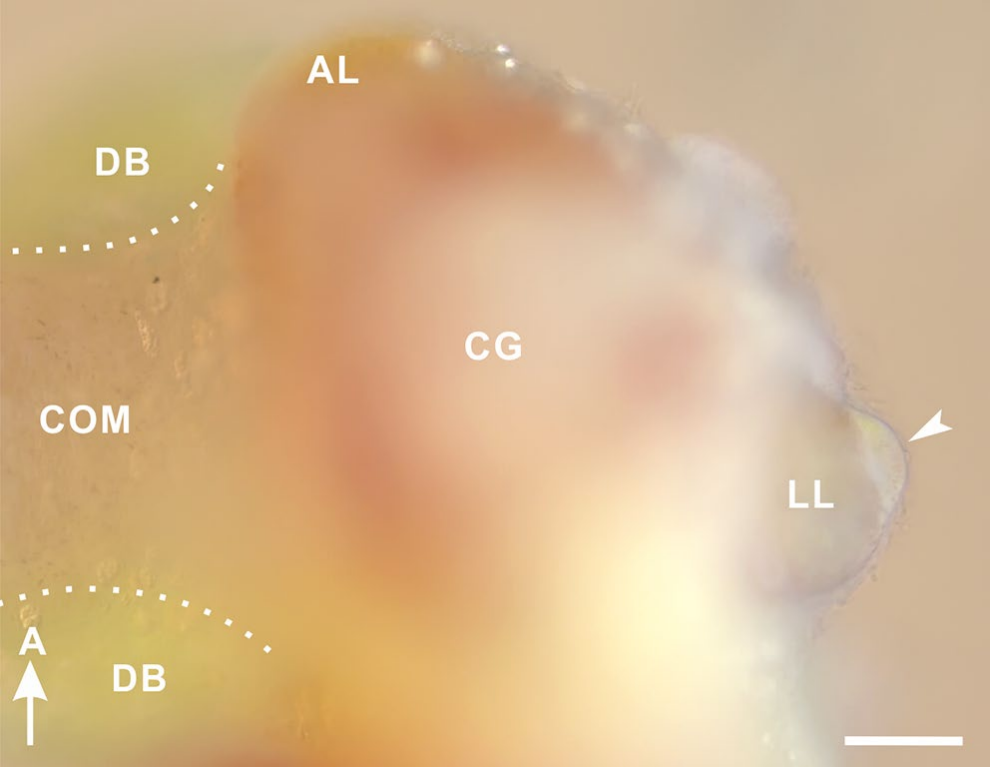
Gross neuroanatomy of a canopy cell in the pond snail, *Lymnaea stagnalis.* Left cerebral ganglion (CG) of a 26-week-old snail reared under long-day conditions (16L8D) at 20 °C (ventral view). A white-orange cell body of a canopy cell (arrowhead) is visible in a lateral side of the lateral lobe (LL), a budding structure of the cerebral ganglion. A, anterior; AL, anterior lobe; COM, commissure; DB, dorsal body. Scale bar: 100 μm

The canopy cell is a large, singly occurring neurosecretory cell in the lateral lobe of the cerebral ganglion; the lateral lobe houses a single canopy cell (arrowhead in Fig. 1). The cell body exhibits a canopy-like shape (Lever and Joosse 1961) and displays white-orange appearance, ranging white to orange, under reflected light (Fig. 1). These features, combined with the extraordinary cell size, allow us to distinguish the canopy cell from the numerous other ordinary neurons and glia within the lateral lobe. The color seems to vary with the number of neurosecretory granules (Benjamin et al. 1980).

### Single morphology of a canopy cell

**Fig. 2:**
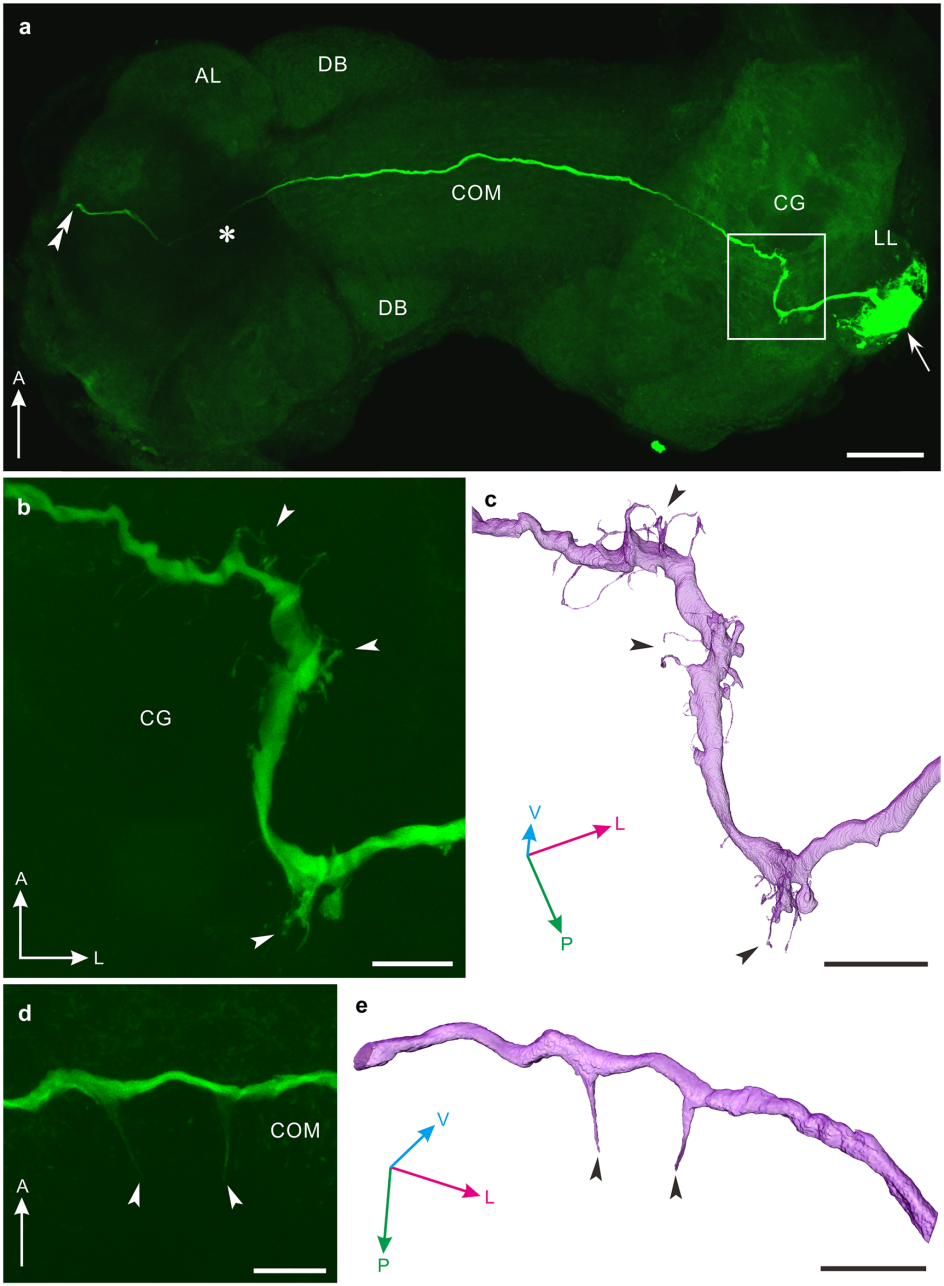
Morphology of a single canopy cell in the pond snail, *Lymnaea stagnalis*. Confocal stack of a single canopy cell (**a**, **b**, **d** ventral view). **a** Cell body of a canopy cell (arrow) in the lateral lobe (LL) sends the primary axon to the ipsilateral cerebral ganglion (CG), where the axon bears many short collaterals. The axon continues to run through the commissure (COM) without bearing any neurites and enters the contralateral cerebral ganglion to leave the ganglion at a point indicated by the double arrowhead. Note that the axon thins in the area marked by an asterisk, but continues to the exit. **b** Enlarged image of the area indicated by the square in panel a. Many short collaterals from the main axon are visible (arrowheads). **c** Three-dimensional reconstruction of the image in panel b, observed from a different angle. The main axon bearing short collaterals (arrowheads). **d**, **e** Pair of fibers extending posteriorly in the commissure (arrowheads) in another preparation. Their smooth appearance is clearly seen in the reconstructed image in panel e. For orientation in panels c and e, refer to three-way arrows in the panels. A, anterior; AL, anterior lobe; DB, dorsal body; L, lateral; P, posterior; V, ventral. Scale bars: **a** 100 μm, **b**, **d** 20 μm, and **c**, **e** 30 μm

We studied the projection pattern of the canopy cell by intracellular dye injection (Fig. 1). A primary axon stemming from the cell body enters the ipsilateral cerebral ganglion and winds there in a characteristic S-shape to project toward the contralateral cerebral ganglion through the intercerebral commissure, which serves as a neurohemal site for neurosecretory cells in the cerebral ganglia (Fig. 2a). The ipsilateral main axon bears many short collaterals (arrowheads in Fig. 2b). The 3D reconstructed image clearly revealed these neurites (arrowheads in Fig. 2c). Most of canopy cells filled with Lucifer yellow run through the commissure without bearing any neurites (Fig. 2a) although it has been reported that they extend characteristic fibers with disc-shaped endings in the commissure (van Minnen et al. 1979).

**Fig. 3:**
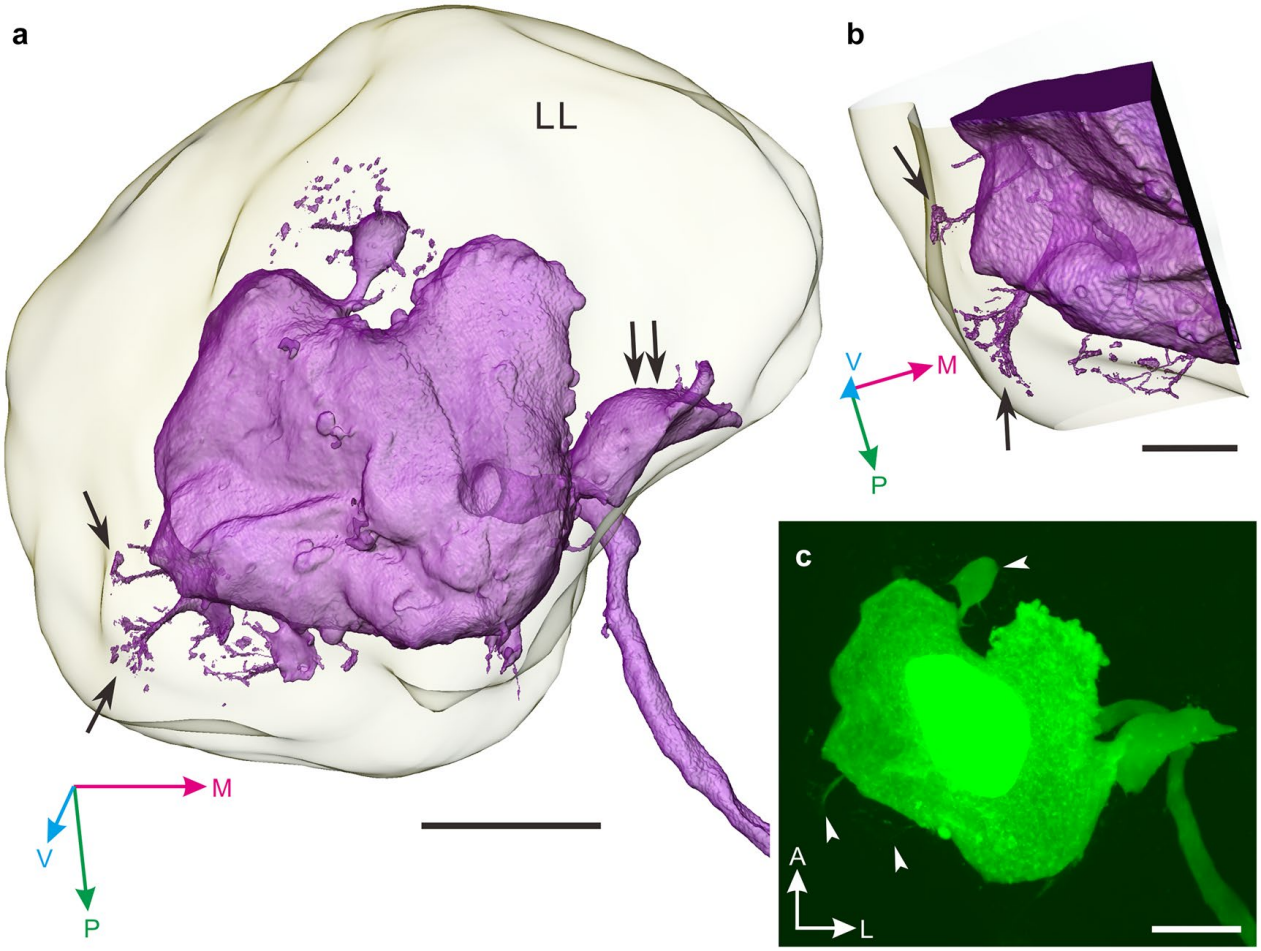
Morphology of a cell body of a canopy cell in the pond snail, *Lymnaea stagnalis*. **a**, **b** Three-dimensional reconstruction of a cell body of a canopy cell, situated in the lateral lobe (LL). Corresponding neurite-like extensions are indicated with arrows. These appear to be attached to the membrane of the lateral lobe (arrows in panel b). Twin arrows in panel a indicate a distinctive large extension stemming from the cell body. **c** Confocal stack of a cell body of a canopy cell filled with Lucifer yellow, which was applied to the 3D reconstruction. The cell body extends some neurite-like structures from the plasma membrane (arrowheads). The fiber-like structures are clearly visible in the reconstructed image in panels a and b. A, anterior; L, lateral; M, medial; P, posterior; V, ventral. Scale bars: **a** 30 μm, **b** 10 μm, **c** 20 μm

In the present study, 31 canopy cells were successfully labeled by intracellular dye injection. The main axon, which invades the contralateral cerebral ganglion, exits the ganglion (double arrowhead in Fig. 2a) to extend further through the median lip nerve and to form terminal arbors on the nerve (not shown). In 2 of the 31 snails studied, we found a pair of smooth fibers extending posteriorly in the commissure (Fig. 2d, e), but neither neurites nor collaterals in the commissure were observed in the other 29 canopy cells. These neurites are, in appearance, completely different from those reported previously (van Minnen et al. 1979). In addition, we imaged the cell body filled with Lucifer yellow by high-magnification confocal microscopy and performed a 3D reconstruction (Fig. 3). The 3D image explicitly revealed that some fibrous structures stem from the cell membrane (Fig. 3a) and appear to attach to inner part of the sheath enveloping the lateral lobe (arrows in Fig. 3b). Interestingly, a large distinctive extension was clearly visible in this particular canopy cell (twin arrow in Fig. 3a).

### Double labeling of a canopy cell and CDCs

**Fig. 4:**
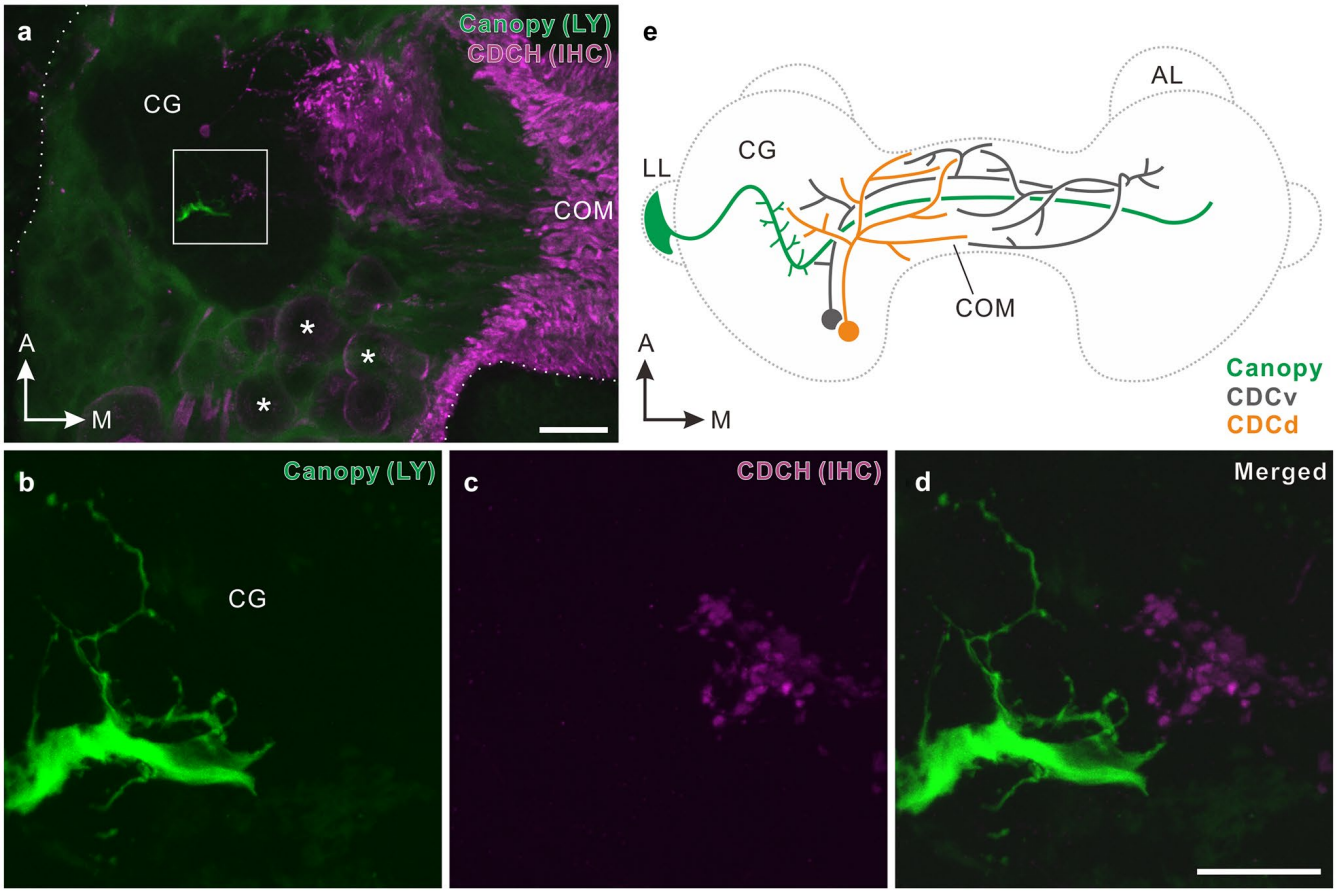
Double labeling of a canopy cell and caudo-dorsal cells in the pond snail, *Lymnaea stagnalis*. **a** Confocal stack of a horizontally sliced micro-section (40 μm thickness) of the cerebral ganglion containing part of a primary axon of a canopy cell filled with Lucifer yellow by intracellular dye injection (green) and caudo-dorsal cells (CDCs) immunohistochemically labeled by anti-CDCH-I (magenta). Asterisks indicate some cell bodies of CDCs. **b**–**d** Double labeling of the canopy cell (green in b) and CDCs (magenta in c), representing the square in panel a. Corresponding merged image in (d). Neurites of the canopy cell and those of CDCs closely locate, but do not overlap. **e** Schematic illustration depicting projection patterns of the canopy cell (green) and CDCs of two morphological types, i.e., ventral CDC (CDCv, gray) and dorsal CDC (CDCd, orange). A, anterior; AL, anterior lobe; COM, commissure; IHC, immunohistochemistry; LL, lateral lobe; LY, Lucifer yellow; M, medial. Scale bars: **a** 50 μm, **d** 20 μm; **b**, **c** and **d** have the same scale

The canopy cell and CDCs appeared to form their neurites in close proximity in the cerebral ganglia in independent preparations. Thus, we evaluated whether they potentially form direct neural connections by a double-labeling method. Figure 4a shows a confocal stack of a micro-slice of the unilateral cerebral ganglion containing both parts of an axon of the canopy cell and CDCH-I-immunoreactive neurites. Ipsilateral collaterals of the canopy cell (Fig. 4b) and varicose neurites of CDCs (Fig. 4c) closely distribute in the cerebral ganglion; however, direct contact was not seen (*n* = 3, Fig. 4d), suggesting that synaptic regulation of CDCs by the canopy cell is unlikely. The distribution pattern of the canopy cell and two types of CDCs (CDCv and CDCd) is illustrated in Fig. 4e.

### Electrophysiological properties of canopy cells in long-day and medium-day conditions

In the present study, we examined photoperiodism in egg laying. At 24 weeks after hatching, the proportion of snails that had undergone egg laying was significantly higher in long-day conditions [100% (*n* = 45)] than in medium-day conditions [14.9% (*n* = 47)] (*p* < 0.001, chi-square test; Supplementary Fig. 1). In long-day conditions, we used snails that laid more than 7 egg masses before intracellular recording had been performed. Contrarily, in medium-day conditions, we used snails that had not undergone egg laying. The shell height of snails in which we succeeded in recording the canopy cell was 21.9 ± 1.3 mm (mean ± SD, *n* = 15) for long-day snails and 22.3 ± 1.0 mm (mean ± SD, *n* = 16) for medium-day snails. We found no significant difference in the shell height between the two photoperiodic conditions (*p* > 0.05, unpaired two-tailed *t*-test).

**Fig. 5:**
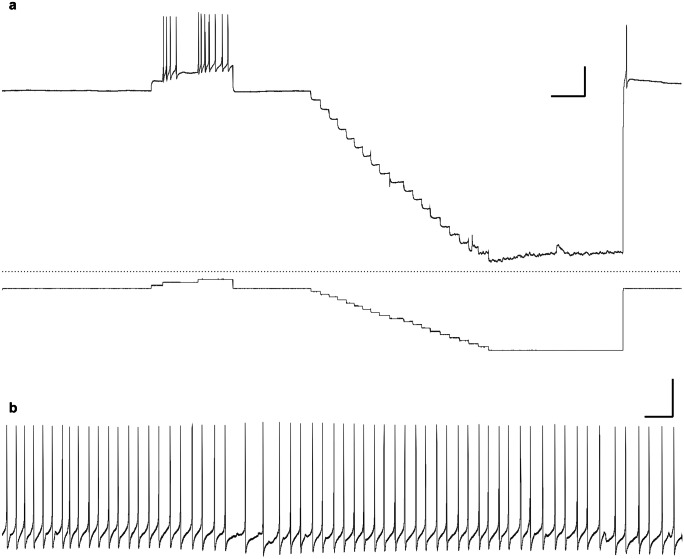
Intracellular recording from a canopy cell in the pond snail, *Lymnaea stagnalis*. **a** Intracellular recording was performed from a cell body of a canopy cell of a 25-week-old snail reared at 20 °C under long-day conditions (16L8D). The resting membrane potential for this particular neuron was − 67.7 mV. It did not show spontaneous action potentials, but spikes were evoked by injection of positive direct current of 0.2 nA. The latter half of the upper trace represents change of the membrane potential caused by stepwise hyperpolarizing current injection. The bottom trace represents current applied to the neuron in 0.1 nA steps. **b** Spontaneous action potentials of a canopy cell of a 25-week-old snail reared at 20 °C under long-day conditions (16L8D). The membrane potential of this particular neuron was − 32.3 mV. Scale bars: **a** 5 s (horizontal) 40 mV (vertical), **b** 5 s (horizontal) 20 mV (vertical)

We successfully recorded from and also labeled 31 canopy cells in total: 16 neurons in medium-day conditions, together with 15 neurons in long-day conditions (Table 1). Most of the canopy cells were electrically silent at the resting membrane potential (Fig. 5a), but generated action potentials after injection of positive current (Fig. 5a). However, 20% of canopy cells in long-day snails (3/15) exhibited spontaneously spiking activities (Fig. 5b); all canopy cells recorded from medium-day snails were silent. There was no significant difference in proportion of occurrence of spontaneously spiking neurons between the two photoperiodic conditions (*p* > 0.05, chi-square test).

**Fig. 6:**
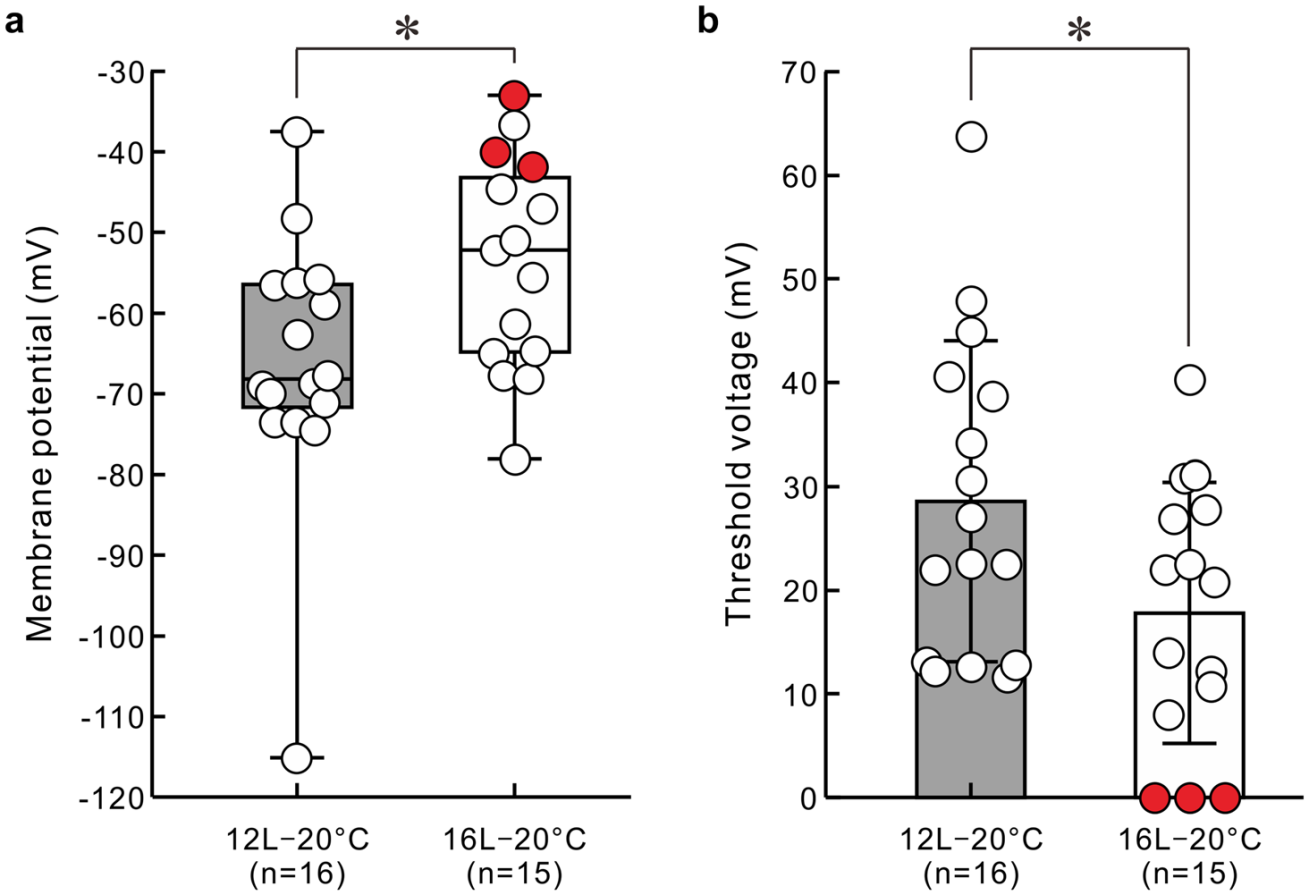
Membrane potential and threshold voltage of a canopy cell in the pond snail, *Lymnaea stagnalis*. **a** Membrane potentials of the canopy cells were significantly deeper in medium-day conditions (12L12D) than in long-day conditions at 20 °C. Box plot indicates median, interquartile ranges, and maximum and minimum values. **b** Threshold voltages of the canopy cells (mean ± SD) were significantly larger in medium-day conditions (12L12D) than in long-day conditions (16L8D) at 20 °C. Red circles represent values of spontaneously spiking neurons. *, *p* < 0.05 (Mann–Whitney *U* test for the membrane potential, and unpaired two-tailed *t*-test for the threshold voltage)

Here, we compared seven electrophysiological properties between two photoperiodic conditions of long-day and medium-day conditions (Fig. 6 and Table 1). We revealed no significant differences in these parameters, other than the membrane potential and threshold voltage, between long-day and medium-day conditions (*p* > 0.05, Mann–Whitney *U* test, or unpaired two-tailed *t*-test; Table 1). Interestingly, the membrane potential was shallower in long-day conditions than in medium-day conditions (*p* < 0.05, Mann–Whitney *U* test; Fig. 6a). Accordingly, the threshold voltage was smaller in long-day conditions than in medium-day conditions (*p* < 0.05, unpaired two-tailed *t*-test; Fig. 6b). It seems that the membrane potentials of spontaneously spiking neurons tended to be shallower compared to the resting membrane potentials of silent neurons (Fig. 6a). Representative current–voltage relationships for 2 canopy cells are shown in Supplementary Fig. 2. The value of the input resistance (Table 1) for each canopy cell was estimated by calculating linear regression of the linear portion of the curve (see “Materials and methods” section for detail).

## Discussion

Egg-laying activity of the pond snail, *L. stagnalis*, is photoperiodically regulated (Bohlken and Joosse 1982; Hamanaka and Shiga 2021), and such regulation appears to be mediated by photoperiodic change of the excitability of CDCs (Hamanaka and Shiga 2021) as well as regulated expression of *cdch* in the CDCs (Kitai et al. 2021). In addition to CDCs, paired lateral lobes in the cerebral ganglia are also known to be involved in accelerating egg-laying activity (Geraerts 1976a). In the present study, we focused on the canopy cell situated in the lateral lobe as one of regulators of CDCs and re-evaluated its spatial projection pattern in relation to that of CDCs. Furthermore, we performed intracellular recording of the canopy cell in both long-day and medium-day conditions, and examined if the neuron changes its electrophysiological properties in a photoperiod-dependent manner.

Anatomical investigation revealed a discrepancy in arborization of the main axon within the commissure between the present and previous accounts; the latter reported that the commissure receives characteristic neurites with disc-like termini (van Minnen et al. 1979); such neurites were not observed in the present study and instead novel posterior fibers were found in a few canopy cells. Further, there were no direct neural connections with the CDCs that trigger ovulation and subsequent egg-laying behavior (de Vlieger et al. 1980; ter Maat et al. 1986, 1989; Geraerts et al. 1991). Finally, intracellular recording has demonstrated that the canopy cell is generally silent and that the electrophysiological properties are only under moderate photoperiodic regulation; the excitability appears to be higher in long-day conditions than in medium-day conditions. Plausible function and photoperiodical regulation of the canopy cell are discussed below.

### Anatomical insight of a canopy cell

In the present study, the projection pattern of the canopy cell in the cerebral ganglia was closely examined, and then we confirmed the ipsilateral collaterals along the main axon as well as the multiple neurite-like extensions stemming from the plasma membrane of the cell body that have been reported in a previous study (Benjamin et al. 1980). Anatomical inspection with an optical microscope does not clearly reveal the function of neuronal structures, although Benjamin et al. (1980) argued that the neurite-like structures are potential release sites of neurosecretory materials due to their shape. We propose the structures might instead have a role as either scaffolding structure to immobilize the cell body or as a sensor to monitor osmotic pressure in the hemolymph. In the pond snail, a previous study argued, based on Gomori staining, that the canopy cell may be involved in osmotic regulation via an anti-diuretic factor since the neurosecretory materials are deprived of the cell body in exposure of snails to salt water whose osmotic pressure is higher than the hemolymph (Lever and Joosse 1961). The extensions of the cell body may function as a sensor to monitor osmotic pressure in the hemolymph and thus maintain water content in the body at an appropriate level. Detailed EM observation of the structure may help us to elucidate its role.

Regarding another anatomical aspect of this neuron, a previous account has reported that the canopy cell bears distinct multiple neurites with a disc-shaped ending in the commissure (van Minnen et al. 1979). However, we could not identify any such fibers. Instead, we identified a pair of smooth neurites in the commissure in a few canopy cells (Fig. 2d, e). Since their appearances rather differ, we distinguished this pair of neurites (Fig. 2d, e) from those of the previous account (van Minnen et al. 1979) and designated the former posterior fibers. The previous report used horseradish peroxidase as a neural tracer, and the enzyme was iontophoretically injected into a soma of a canopy cell of somewhat younger snails, i.e., 5 months old (shell height: 25–30 mm) (van Minnen et al. 1979). Since the molecular weight of horseradish peroxidase is larger than that of the Lucifer yellow, it is unlikely that injected Lucifer yellow failed to spread into the neurites with disc-shaped ending in the commissure (Fig. 2). The observation of the characteristic fibers in the commissure (van Minnen et al. 1979) might be attributed to simultaneous penetration of more than two neurons in the lateral lobe to create a pseudo-neuron morphology. Only in a few snails does the canopy cell extend the paired posterior fibers within the commissure, which does not favor the possibility that the neurons release the neurosecretory materials from the commissure into the hemolymph at least in snails 25 to 26 weeks old.

In addition to analyzing the morphology of the single canopy cell, we examined spatial relationship of both the neurites of the canopy cell and those of CDCs in the cerebral ganglia because they appeared to locate in close proximity. The lateral lobe activates CDCs and also accelerates egg-laying activity (Geraerts 1976a; Roubos et al. 1980), and CDCs trigger the egg-lying behavior via an orchestrated long-lasting discharge and subsequent release of CDCH-I (de Vlieger et al. 1980; ter Maat et al. 1986, 1989). Given that the canopy cell is responsible for the activation of CDCs, we thought that the canopy cell might be presynaptic to CDCs. However, double labeling showed no evidence of overlapping of the two types of NSCs. It seems that synaptic regulation of CDCs by the canopy cell is unlikely, but we cannot exclude the possibility that the canopy cell signals humorally via release of secretory materials to CDCs.

Nevertheless, both the canopy cell and CDCs project the neurites close together in the cerebral ganglion, and thus they may receive inputs from a set of neurons of the same kind. Detailed anatomical inspection of the corresponding area may help to identify and characterize the local circuits underlying photoperiodic regulation of neuroendocrine systems in *L. stagnalis*.

### General electrophysiological properties of the canopy cell

A previous study showed the possibility that bilateral canopy cells might be electrically coupled by simultaneous intracellular recording, but the authors did not examine the electrophysiological properties in any detail (Benjamin and Rose 1984). In the present study, we found that most canopy cells recorded were silent, with resting membrane potentials ranging from − 37 to − 78 mV. Steady intracellular current injection exceeding the threshold generated action potentials with a spike height of 40 to 87 mV and a spike duration of 12 to 34 ms at half amplitude. However, 20% of canopy cells in long-day snails (3/15) spontaneously generated action potentials (Fig. 5b), the electrophysiological properties of which were almost the same as those elicited by positive current injection. Excitability of the canopy cells is relatively higher in long-day conditions (Fig. 6), and it may vary depending on either internal or external signal inputs.

### Comparison of medium-day and long-day snail’s canopy cells

A previous quantitative EM study revealed that subcellular organelles indispensable for the production of secretory materials, such as the rough endoplasmic reticulum and Golgi zone, are significantly larger in volume under long-day conditions than under short-day conditions (van Minnen and Reichelt 1980). Since the secretory activity of neurons is generally correlated with electrophysiological properties, it is most likely that those of the canopy cell are photoperiodically controlled.

The comparison between two photoperiodic conditions determined that the membrane potential is shallower in long-day conditions than in medium-day conditions, and that the threshold voltage is smaller in long-day conditions than in medium-day conditions (Fig. 6). However, there is no significant difference in the membrane potential for 1st spike generation between the two photoperiodic conditions (Table 1). In long-day conditions, we found that some canopy cells generate action potentials spontaneously, but there was no significant difference in ratio of occurrence of the spontaneously spiking neurons between the two photoperiodic conditions. In long-day conditions, the resting membrane potential may shift toward the threshold value, and the canopy cell will generate action potentials spontaneously if the resting membrane potential reaches the threshold. Alternatively, short- or medium-day information may cause the resting membrane potential to be hyperpolarized. Possibly, both regulatory mechanisms occur simultaneously. Additionally, in the present study, we cannot rule out the possibility that egg laying might affect activity of canopy cells in long-day conditions. Essential role of membrane potential in the activity regulation is supported by the observation that the membrane potentials of spontaneously spiking neurons are shallow compared to those of silent neurons (Fig. 6a). Photoperiodic regulation of the resting membrane potential has also been reported in CDCs (Hamanaka and Shiga 2021) and a type of brain NSCs in the tobacco horn moth (Tomioka et al. 1995). Regulated expression of ion channels might be responsible for regulation of the resting membrane potentials (*vide infra*).

Recent transcriptome analysis in the pond snail CNS has identified several ion channels (Dong et al. 2021). These include Na^+^, K^+^, Ca^2+^, cation, Cl^−^, and transient receptor potential (TRP) channels (Dong et al. 2021). Of these ion channels, those conducting leak current are essential in establishing the resting membrane potential (Hodgkin and Huxley 1947; Lu and Feng 2011), which plays an important role in excitability of neurons. For instance, in the right pedal dorsal 1 neuron of the pond snail, which is one of the neurons comprising a central pattern generator for aerial respiration, RNAi-mediated knockdown of U-type channels conducting Na^+^ leak current hyperpolarizes the neuron and disrupts the rhythmic action potentials, resulting in suppression of aerial respiratory behavior (Lu and Feng 2011).

In the hypoglossal motoneuron and cerebellar granule neuron of rats, a two-pore domain potassium leak channel (TASK-1), which generates outward current in an extracellular pH-dependent manner, regulates the membrane potential as well as the activity of the neurons (Millar et al. 2000; Talley et al. 2000). A homologous TASK channel (LyTASK) has also been characterized in the pond snail (Andres-Enguix et al. 2007), and thus it is plausible that up-regulated expression of LyTASK hyperpolarizes the canopy cell. Photoperiodically controlled expression of these ion channels may be responsible for the regulation of the resting membrane potential in the canopy cell. It is waited to explore whether the neuron expresses these ion channels or not. Given molluskan NSCs are amenable to transcriptome analysis at the single cell level due to the large cell size (Moroz and Kohn 2013), it would be feasible to quantitatively compare the expression of a number of ion channel genes between different photoperiodic conditions. Whether and how the expression of such ion channels changes photoperiodically is a subject for future research.

### Plausible function of the canopy cell and future perspectives

The canopy cell had been identified in the 1960s (Lever and Joosse 1961), and its axon terminals were found to be filled with neurosecretory granules by EM study (Brink and Boer 1967) and to express MIPs (van Minnen et al. 1989; Meester et al. 1992; Smit et al. 1992, 1998). However, other than a potential role in osmotic regulation (Lever and Joosse 1961), its function remains to be unraveled.

The lateral lobe, which houses the canopy cell, has been demonstrated to inhibit LGCs while the lobe stimulates CDCs as well as the dorsal body, the latter being an endocrine organ surmounting the cerebral ganglia and producing gonadotropic factor (Geraerts 1976a; Roubos et al. 1980). Although responsible cells for those functions have not been identified yet, the following reports support that the canopy cell is the one controlling both LGCs and CDCs. The main axon of the canopy cell invades the ipsilateral cerebral ganglion and runs along those of the LGCs, which promote body growth (Geraerts 1976b; Smit et al. 1998), and thus retardation of the growth might be accomplished by neural connections between the canopy cell and LGCs. Indeed, body growth is attenuated by long-day conditions (Bohlken and Joosse 1982; Kitai et al. 2021), in which the canopy cell displays higher excitability than in medium-day conditions. Additionally, the canopy cell may activate the CDCs and dorsal body via its neurosecretory materials, which subsequently accelerates both egg production and egg laying. Taken together, the canopy cell in the lateral lobe appears to be dedicated to coordinate between reproduction and body growth; for example, the canopy cell might accelerate egg laying at the expense of growth under long-day conditions, whereas it might prompt body growth at the expense of reproduction under medium-day conditions.

The canopy cell has a distinctive large cell body, which provides direct accessibility under a dissecting microscope. Further, the pond snail is known to be rather tolerant to surgical ablation (Geraerts 1976a, b; Roubos et al. 1980). These features should allow us to assess roles of the canopy cell in reproduction and body growth through the observation of snails with the canopy cells surgically ablated. Our findings will be instructive in understanding the photoperiodically regulated coordination between reproduction and body growth in the pond snail *L. stagnalis*.

## Supplementary Information

Below is the link to the electronic supplementary material.

Supplementary file1: Cumulativeproportion of egg-laying pond snails, *Lymnaeastagnalis.* Horizontal axis indicates weeks after hatching and vertical axisthe cumulative proportion of the snails that had undergone egg laying at leastonce. Closed circles indicate results from the snails reared under medium-dayconditions (12L12D) at 20 °C, whileopen circles indicate those under long-day conditions (16L8D) at 20 °C. From17 weeks after hatching,the cumulative proportion of egg-laying snails was significantly higher inlong-day conditions than in medium-day conditions (*, *p* < 0.05; ***, *p* < 0.001;chi-square test). The maximum difference between the two photoperiodicconditions was observed 22 weeksafter hatching.* N.S.*, no significantdifference (TIF 5676 KB)

Supplementary file2: Representativecurrent–voltage relationship of acanopy cell in the pond snail, *Lymnaeastagnalis*. **a** Recording wasperformed on a 25-week-old snail reared at 20 °Cunder medium-day conditions (12L12D). Input resistance was estimated bycalculating the liner regression through the linear portion of the current–voltage relation. The estimated inputresistance value for this neuron was 127.46 MΩ. **b**Recording was performed on a 25-week-old snail reared at 20 °C under long-day conditions (16L8D).The estimated input resistance value for this neuron was 113.51 MΩ (TIF 6886 KB)

## Data Availability

The data that support the findings of this study are available from the corresponding author upon reasonable request.
