# Supplementary figures and images for: Unique morphology and photoperiodically regulated activity of neurosecretory canopy cells in the pond snail Lymnaea stagnalis
Source: Cell Tissue Res. 2023 Jul 7;393(3):547–58. doi: 10.1007/s00441-023-03799-x (PMC10484813; doi:10.1007/s00441-023-03799-x)

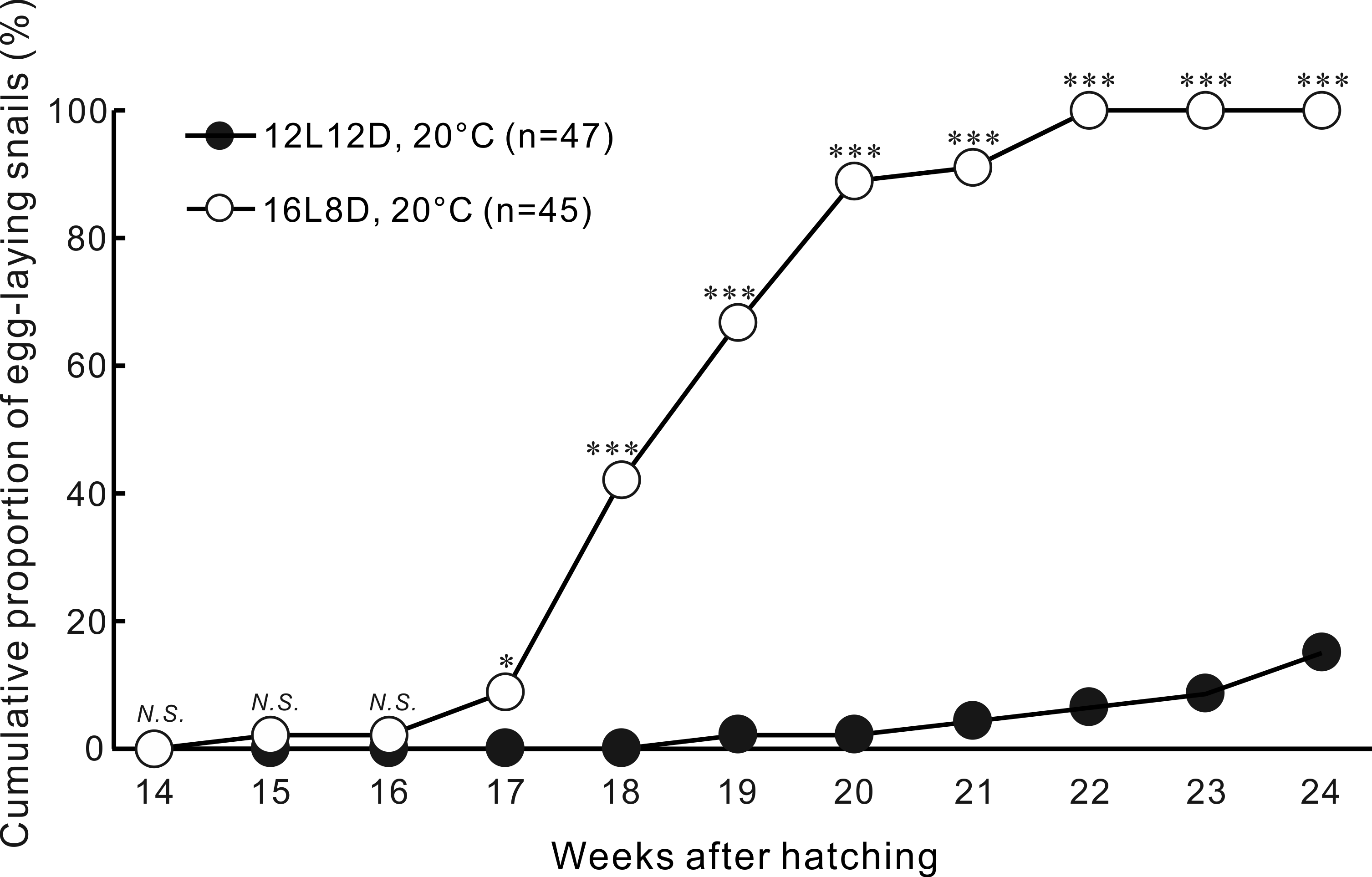

Supplement: Supplementary file 1 — Supplementary file1: Cumulativeproportion of egg-laying pond snails, Lymnaeastagnalis. Horizontal axis indicates weeks after hatching and vertical axisthe cumulative proportion of the snails that had undergone egg laying at leastonce. Closed circles indicate results from the snails reared under medium-dayconditions (12L12D) at 20 °C, whileopen circles indicate those under long-day conditions (16L8D) at 20 °C. From17 weeks after hatching,the cumulative proportion of egg-laying snails was significantly higher inlong-day conditions than in medium-day conditions (*, p < 0.05; ***, p < 0.001;chi-square test). The maximum difference between the two photoperiodicconditions was observed 22 weeksafter hatching. N.S., no significantdifference (TIF 5676 KB) [file 441_2023_3799_MOESM1_ESM.tif]

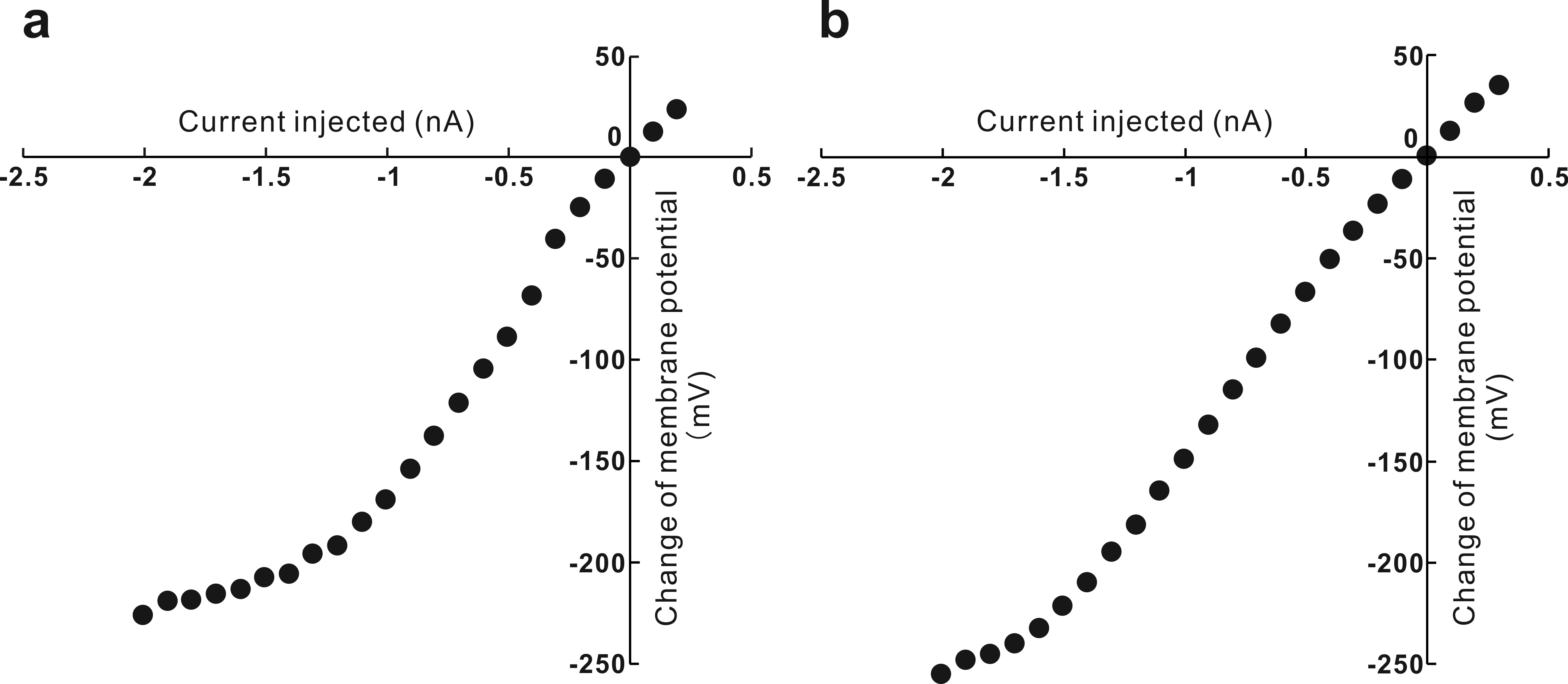

Supplement: Supplementary file 2 — Supplementary file2: Representativecurrent–voltage relationship of acanopy cell in the pond snail, Lymnaeastagnalis. a Recording wasperformed on a 25-week-old snail reared at 20 °Cunder medium-day conditions (12L12D). Input resistance was estimated bycalculating the liner regression through the linear portion of the current–voltage relation. The estimated inputresistance value for this neuron was 127.46 MΩ. bRecording was performed on a 25-week-old snail reared at 20 °C under long-day conditions (16L8D).The estimated input resistance value for this neuron was 113.51 MΩ (TIF 6886 KB) [file 441_2023_3799_MOESM2_ESM.tif]
